# *CDH1* Genotype Exploration in Women With Hereditary Lobular Breast Cancer Phenotype

**DOI:** 10.1001/jamanetworkopen.2024.7862

**Published:** 2024-04-23

**Authors:** Giovanni Corso, Elena Marino, Cristina Zanzottera, Carla Oliveira, Loris Bernard, Debora Macis, Joana Figueiredo, Joana Pereira, Patrícia Carneiro, Giulia Massari, Massimo Barberis, Alessandra Margherita De Scalzi, Sergio Vincenzo Taormina, Elham Sajjadi, Claudia Sangalli, Sara Gandini, Oriana D’Ecclesiis, Cristina Maria Trovato, Anna Rotili, Filippo Pesapane, Luca Nicosia, Carlo La Vecchia, Viviana Galimberti, Elena Guerini-Rocco, Bernardo Bonanni, Paolo Veronesi

**Affiliations:** 1 Division of Breast Surgery, European Institute of Oncology (IEO), Istituto di Ricovero e Cura a Carattere Scientifico (IRCCS), Milan, Italy; 2 Department of Oncology and Hemato-Oncology, University of Milan, Milan, Italy; 3 Clinic Unit of Oncogenomics, IEO, IRCCS, Milan, Italy; 4 Division of Cancer Prevention and Genetics, IEO, IRCCS, Milan, Italy; 5 Instituto de Investigação e Inovação em Saúde, University of Porto, Porto, Portugal; 6 Faculty of Medicine, University of Porto, Porto, Portugal; 7 Institute of Molecular Pathology and Immunology of the University of Porto, Porto, Portugal; 8 Division of Pathology, IEO, IRCCS, Milan, Italy; 9 Data Management, IEO, IRCCS, Milan, Italy; 10 Department of Experimental Oncology, IEO, IRCCS, Milan, Italy; 11 Division of Endoscopy, IEO, IRCCS, Milan, Italy; 12 Division of Breast Imaging, IEO, IRCCS, Milan, Italy; 13 Department of Clinical Sciences and Community Health, Branch of Medical Statistics, Biometry and Epidemiology “G.A. Maccacaro,” University of Milan, Milan, Italy

## Abstract

**Importance:**

Pathogenic or likely pathogenic (P/LP) germline *CDH1* variants are associated with risk for diffuse gastric cancer and lobular breast cancer (LBC) in the so-called hereditary diffuse gastric cancer (HDGC) syndrome. However, in some circumstances, LBC can be the first manifestation of this syndrome in the absence of diffuse gastric cancer manifestation.

**Objectives:**

To evaluate the frequency of germline *CDH1* variants in women with the hereditary LBC (HLBC) phenotype, somatic *CDH1* gene inactivation in germline *CDH1* variant carriers’ tumor samples, and the association of genetic profiles with clinical-pathological data and survival.

**Design, Setting, and Participants:**

This single-center, longitudinal, prospective cohort study was conducted from January 1, 1997, to December 31, 2021, with follow-up until January 31, 2023. Women with LBC seen at the European Institute of Oncology were included. Testing for germline *CDH1*, *BRCA1*, and *BRCA2* genes was performed. Somatic profiling was assessed for germline *CDH1* carriers.

**Main Outcomes and Measures:**

Accurate estimates of prevalence of germline *CDH1* variants among patients with HLBC and the association of somatic sequence alteration with HLBC syndrome. The Kaplan-Meier method and a multivariable Cox proportional hazards regression model were applied for overall and disease-free survival analysis.

**Results:**

Of 5429 cases of primary LBC, familial LBC phenotype accounted for 1867 (34.4%). A total of 394 women with LBC were tested, among whom 15 germline *CDH1* variants in 15 unrelated families were identified. Among these variants, 6 (40.0%) were P/LP, with an overall frequency of 1.5% (6 of 394). Of the 6 probands with P/LP *CDH1* LBC, 5 (83.3%) had a positive family history of BC and only 1 (16.7%) had sporadic juvenile early-onset LBC. No germline *BRCA1* and *BRCA2* variants were identified in *CDH1* carriers. An inactivating *CDH1* mechanism (second hit) was identified in 4 of 6 explored matched tumor samples (66.7%) in P/LP germline carriers. The P/LP *CDH1* LBC variant carriers had a significantly lower age at diagnosis compared with the group carrying *CDH1* variants of unknown significance or likely benign (42.5 [IQR, 38.3-43.0] vs 51.0 [IQR, 45.0-53.0] years; *P* = .03).

**Conclusions and Relevance:**

In this cohort study, P/LP germline *CDH1* variants were identified in individuals not fulfilling the classic clinical criteria for HDGC screening, suggesting that identification of these variants may provide a novel method to test women with LBC with early age at diagnosis and/or positive family history of BC.

## Key Points

**Question:**

What is the frequency of germline *CDH1* variants in hereditary lobular breast cancer (HLBC) predisposition?

**Findings:**

In this cohort study of 394 women with LBC, 15 germline *CDH1* variants were identified in 15 families with HLBC; 40.0% were pathogenic or likely pathogenic (P/LP). The overall frequency of P/LP *CDH1* variants was 1.5% and was associated with age of 45 years or younger at LBC diagnosis and positive family history of BC.

**Meaning:**

The identification of P/LP germline *CDH1* variants in young women with LBC with (or without) family history of BC, not fulfilling the classic *CDH1* genetic screening criteria, may provide an indication to test for *CDH1* gene.

## Teaser

This cohort study examines the frequency of germline *CDH1* sequence variations in women with the hereditary lobular breast cancer phenotype and the association of genetic profiles with clinical-pathological data and survival.

## Introduction

Hereditary lobular breast cancer (HLBC) is a rare inherited cancer predisposition syndrome associated with germline pathogenic or likely pathogenic (P/LP) variants in the *CDH1* gene, in which LBC is the first manifestation without a clear family history of diffuse gastric cancer (DGC).^1^ In 1999, a germline *CDH1* variant was defined as the hallmark of hereditary DGC (HDGC)^2^; further research identified P/LP germline *CDH1* variants also in women with LBC but without clinical evidence of DGC.^3,4^ Initially excluding LBC, the International Gastric Cancer Linkage Consortium (IGCLC) suggested 4 revisions of the clinical criteria for *CDH1* genetic testing,^2,5,6,7^ and at the 2020 IGCLC meeting, LBC was considered a pivotal cancer index for *CDH1* genetic testing independently from DGC manifestation. The IGCLC LBC-oriented criteria recommend to test for *CDH1* in patients with (1) 2 or more independent cases of LBC in the same family at younger than 50 years and (2) bilateral LBC, diagnosed at younger than 70 years (both with no cases of DGC).^7^ In 2018, some of our group established a panel of experts to provide recommendations for HLBC management, and after revisiting retrospective literature data, additional clinical criteria were proposed for *CDH1* genetic testing in women with LBC.^1^ In sporadic LBC, the frequency of germline *CDH1* variant detection is approximately 0.2% to 0.5%,^8^ but in the setting of the HLBC phenotype, the overall frequency is not well established due to a lack of large, prospective cohort studies.^9^

In the current study, we tested a large series of women with diagnosed LBC fulfilling the HLBC clinical criteria, aiming to assess the frequency of germline variants in the *CDH1* gene, genomic inactivation in matched tumor samples, and disease-free and overall survival. The *BRCA1* and *BRCA2* genes were also tested to verify a possible association (or exclusion) between *CDH1* HLBC and the hereditary breast-ovarian cancer syndromes in these families.

## Methods

### Ethics Statement

This cohort study was approved by the European Institute of Oncology ethical committee, and all available participants gave their written consent to be included in the study. The study was conducted in accordance with the Strengthening the Reporting of Observational Studies in Epidemiology (STROBE) reporting guideline.

### Study Participants

For this longitudinal, prospective cohort study, a genetic analysis of the *CDH1*, *BRCA1*, and *BRCA2* genes was conducted from January 1, 1997, to December 31, 2021, on blood samples from selected women with LBC at the European Institute of Oncology. Clinical criteria for genetic testing are described in eTable 1 in Supplement 1. Personal and family history along with clinical and histopathological data were collected in a dedicated institutional database. Available biologic samples (whole blood and tumor specimens) were stored in our institutional biobank. Follow-up was conducted using medical records, pathology reports, and telephone consultations conducted by dedicated personnel. The end of the follow-up period for outcome ascertainment was January 31, 2023. Genetic and psychological counseling was offered to all patients with variants in the *BRCA1*, *BRCA2*, or *CDH1* gene.

### Library Preparation and Next-Generation Sequencing

Genomic DNA was extracted from peripheral blood samples using a MagCore Super Automated Nucleic Acid Extractor (Diatech), and for next-generation sequencing library preparation, DNA was quantified using the Qubit dsDNA HS Assay Kit with the Qubit 3.0 Fluorometer (Life Technologies) following the manufacturer’s instructions. Available samples were analyzed with the Hereditary Cancer Solution CE-IVD multigene panel by SOPHiA Genetics. Library preparation was optimized for 200 ng of total genomic DNA (Qubit quantification) using an enrichment protocol (version PM_T1_5.1.5_r2en July 2017). Libraries were quantified through the 4200 TapeStation (Agilent) and Qubit 3.0 Fluorometer (Life Technologies) and diluted to 4 nM. Following denaturation, a 10-pM dilution was loaded on the Illumina MiSeq System with 3% PhiX Control using the MiSeq, version 2 standard reagent kit and 2 × 250 cycles. The results were retrieved and analyzed in the dedicated platform SOPHiA DDM, which allows for the accurate detection of single nucleotide variations, insertions or deletions, and copy number variations.

### Germline Variant Classification

The identified genetic variants were divided into 5 classes according to the International Agency for Research on Cancer recommendations.^10^ Variant pathogenicity was assessed using the ClinVar database,^11^ the Leiden Open Variation Database,^12^ the Clinical Genome Resource *CDH1* Variant Curation Expert Panel,^13^ and the BRCA Exchange^14^ for *BRCA1* and *BRCA2* genes following the American College of Medical Genetics and Genomics guidelines.^15^ In an attempt to assess the pathogenicity of germline *CDH1* missense variants identified in this study, we also used predictive in silico models, such as PROVEAN, SIFT, PolyPhen, and FoldX (details are provided in eTable 2 in Supplement 1).^16,17,18^

### Tumor Genomic Profiling

Next-generation sequencing analysis of primary LBC tumor samples from *CDH1* germline variant carriers was performed to investigate the potential genetic-based mechanism of E-cadherin inactivation (ie, second hit), including second *CDH1* gene somatic variants and/or loss of heterozygosity (LOH), and to explore the genomic landscape of these tumors. In brief, 10 unstained sections were cut from representative formalin-fixed, paraffin-embedded tumor tissue blocks retrieved from the archives of the Division of Pathology of the European Institute of Oncology and submitted to the FoundationOne CDx assay (Foundation Medicine) according to FoundationOne CDx specimen instructions.

### Intragenic Loss of Heterozygosity and Somatic *CDH1* Promoter Methylation Analyses

For intragenic LOH analysis, we used the following intragenic markers: promoter c.-161C>A transversion (rs16260), silent substitution c.2076T>C at exon 13 (rs1801552), and c.*54C>T polymorphism (rs1801026). *CDH1* promoter methylation analysis was carried out 160 base pairs upstream of the translation start site, encompassing 17 CpG sites. Both protocols were described in detail previously.^19^

### E-Cadherin Immunohistochemistry

E-cadherin immunoreactivity was evaluated using the standard protocol on tumor and normal tissues. We considered the predominant expression pattern as normal (complete membrane staining), aberrant (cytoplasmic and heterogeneous staining), or absent (no staining).

### Statistical Analysis

For continuous variables, the median and IQR were reported, and absolute and relative frequencies were assessed as summary measures of categorical variables. According to the nature of variables, χ^2^ tests and Kruskal-Wallis tests were performed to investigate the associations between germinal variant status and characteristics of patients and tumors. The Mann-Whitney *U* test was used to compare ages between probands and family members. Disease-free survival was estimated using the Kaplan-Meier method, and survival distributions were compared using the log-rank test. A multivariable Cox proportional hazards regression model was used to determine the independent association of germinal variant status with cancer progression or death, adjusting for age. The significance level was set at a global 2-tailed *P* < .05 for all analyses. The statistical analyses were performed with RStudio, version 4.2.3 (RStudio).

## Results

### Germline Genetic Testing Results

**Figure 1.**
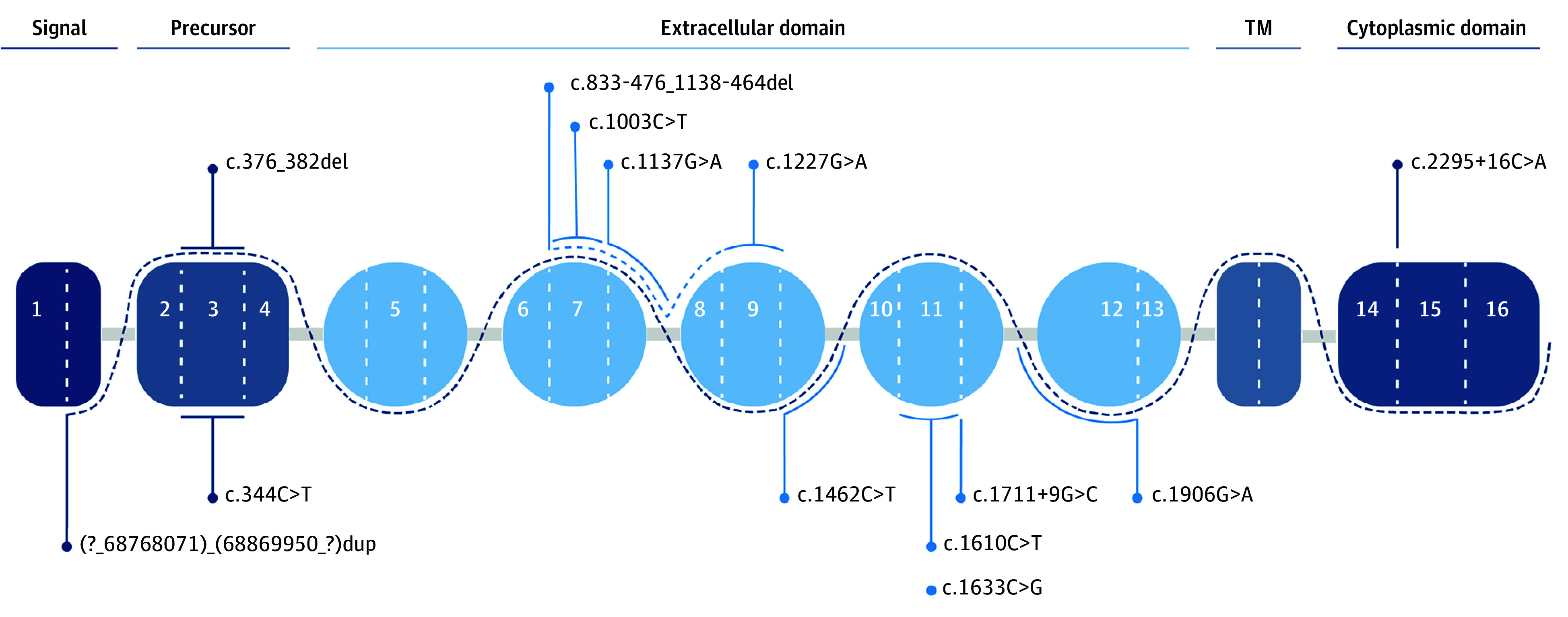
Localization of *CDH1* Variants Identified in Our Study Germline pathogenic or likely pathogenic variants are given below the dashed line, and variants of unknown significance or benign or likely benign variants are given above the dashed line. TM indicates transmembrane.

From an initial consecutive population of 5429 cases of primary LBC, we selected 1867 patients with LBC using the HLBC clinical criteria (eTable 1 in Supplement 1). We were able to enroll 421 women with LBC, of whom 394 (93.6%) were actually tested. Twenty-seven individuals (6.4%) eventually refused to participate or were not available to provide informed consent (eFigure 1 in Supplement 1). All women with LBC were White and from the Italian geographic area. Familial LBC phenotype accounted for 1867 of the 5429 cases (34.4%) (eFigure 1 in Supplement 1). For *CDH1*, 15 of the 394 index cases (3.8%) that met previous^1^ and new, expanded criteria (including early-onset sporadic LBC at age 45 years or younger) were found to have a germline heterozygous *CDH1* variant. Thirteen distinct variants were found (Figure 1); c.1003C>T and c.1633C>G variants were identified in 2 unrelated index cases. Six of the 15 variants (40.0%) were classified as P/LP. The overall frequency of identified P/LP *CDH1* variants was 1.5% (6 of 394) (Table 1), and the variants were identified only in the invasive LBC histotype. Pathogenic or likely pathogenic *BRCA1* and *BRCA2* occurred with a frequency of 0.2% and 1.2%, respectively. Missense variants were also evaluated using in silico tools, and the results are reported in eTable 2 in Supplement 1. Lastly, no co-occurrence of germline *BRCA1* and *BRCA2* variants (eTables 3 and 4 in Supplement 1) was observed in any germline *CDH1* variant carriers (Table 1).

**Table 1.** Germline *CDH1* Variants Identified in 15 Patients With LBC

Proband identification No.	Age, y	Sequence variation^a^	Protein change	Type	Localization	Interpretation
834-001	52	c.1906G>A	p.(Ala636Thr)	Missense	Exon 12	VUS
834-003	43	c.1003C>T	p.(Arg335Ter)	Nonsense	Exon 7	P/LP
834-016	43	c.2295+16C>A	(p.?)	Intronic	Intron 14	B/LB
834-042	63	c.1462C>T	p.(Pro488Ser)	Missense	Exon 10	VUS
834-059	44	c.376_382del	p.(Pro126IlefsTer87)	Frameshift	Exon 3	P/LP
834-096	37	c.1137G>A	p.(Thr379 = )	Synonymous	Exon 8	P/LP
834-155	54	c.1610C>T	p.(Pro537Leu)	Missense	Exon 11	VUS
834-163	53	c.1711+9G>C	(p.?)	Intronic	Intron 11	B/LB
834-173	36	c.833-476_1138-464del	p.(Gly278ValfsTer7)	Deletion	Exon 7-8	P/LP
834-183	39	c.1633C>G	p.(Arg545Gly)	Missense	Exon 11	VUS
834-190	45	c.344C>T	p.(Thr115Met)	Missense	Exon 3	VUS
834-263	43	c.1227G>A	p.(Trp409Ter)	Nonsense	Exon 9	P/LP
834-269	50	(?_68768071)_(68869950_?)dup	NA	CNV	Exon 1-16	VUS
834-323	41	c.1633C>G	p.(Arg545Gly)	Missense	Exon 11	VUS
834-369	43	c.1003C>T	p.(Arg335Ter)	Nonsense	Exon 7	P/LP

Abbreviations: B/LB, benign or likely benign; CNV, copy number variation; LBC, lobular breast cancer; NA, not applicable; P/LP, pathogenic or likely pathogenic; VUS, variant of unknown significance.

^a^ Human Genome Variation Society nomenclature (reference sequence [Human Feb. 2009 - GRCh37/hg19 Assembly]: NM_004360.51).

### Pedigree Features of Germline *CDH1* Variant Carriers

The reported germline *CDH1* variants were identified in 15 unrelated families. Excluding c.1137G>A and c.833-476_1138-463del alterations,^20^ the remaining identified *CDH1* variants in this study were not previously reported in patients with LBC, to our knowledge.^21^ The identified P/LP *CDH1* variants were associated with a positive family history for BC in 5 cases (83.3%; 4 early-onset LBC at age <45 years at diagnosis, 1 also with bilateral LBC manifestation), and 1 (16.7%) case was a sporadic juvenile early-onset LBC (eFigure 2 in Supplement 1).

### Somatic *CDH1* Genetic and Epigenetic Alterations

Data from tumor genomic testing were obtained for 12 of the 15 *CDH1* germline variant carriers (80.0%). No tumor samples were available for 2 cases (13.3%) (1 benign or likely benign [B/LB] [6.7%] and 1 variant of unknown significance [VUS] [6.7%]); 1 tumor specimen (P/LP) did not yield sufficient DNA for the analysis (6.7%). Eleven of 12 explored samples (91.7%) manifested at least a somatic alteration (7 of 12 [58.3%], sequence variations; 6 of 12 [50.0%], intragenic LOH; 3 of 12 [25.0%], promoter methylation) (Table 2 and Table 3). In 5 of the 12 samples (41.7%), we aimed to explore the second-hit mechanism and, in 7 samples (58.3%), somatic inactivation. In P/LP germline *CDH1* carriers, an inactivating *CDH1* mechanism (second hit) was identified in 4 of 6 explored matched tumor samples (66.7%). In this group, we also identified the germline variants in matched tumor specimens but not additional *CDH1* somatic variants. Intragenic LOH was the most common inactivating second-hit mechanism (3 of 5 cases [60.0%]), and methylation was identified in only 1 of 5 cases (20.0%) (Table 2).

**Table 2.** Identified Somatic Alterations, or Second Hit, in 6 Patients With HLBC With Germline P/LP *CDH1* Variant

Proband identification No.	HLBC *CDH1* variant	Tumor				Structural		Epigenetic, methylation	E-cadherin IHC expression	Second-hit *CDH1*	Other P/LP variants
		Germline *CDH1* variant detected	VAF, %	Somatic *CDH1* variant detected	VAF, %	Variant	iLOH				
834-003	c.1003C>T	Yes	82.4	No	No	No	Yes	No	Negative	iLOH	*AKT3*, *MDM4*, *IKBKE*, *PARP1*, *PIK3C2B*
834-059	c.376_382del	Yes	77.2	No	No	No	Yes	No	Negative	iLOH	*CCND1*, *C11ORF30*, *FGF19*, *FGF3*, *FGF4*
834-096	c.1137G>A	NA	NA	NA	NA	NA	NA	NA	NA	NA	NA
834-173	c.833-476_1138-464del	Yes	CNV	No	No	No	Yes	No	Negative	iLOH	*PIK3CA*, *MDM4*, *GATA3*, *PIK3C2B*
834-263	c.1227G>A	Yes	70.6	No	No	No	No	No	Negative	Not found	*PIK3CA*, *CCND1*, *C11ORF30*
834-369	c.1003C>T	Yes	58.5	No	No	No	No	Yes	Negative	Methylation	*ERBB2*, *PTEN*, *BRD4*, *NOTCH3*, *PRKAR1A*, *SMARCA4*

Abbreviations: CNV, copy number variation; HLBC, hereditary lobular breast cancer; IHC, immunohistochemistry; iLOH, intragenic loss of heterozygosity; NA, unavailable tumor sample; P/LP, pathogenic or likely pathogenic; VAF, variant allele frequency.

**Table 3.** Overall Somatic Alteration in 9 Sporadic LBCs With Germline VUS or LB *CDH1* Germline Variant Carrier

Proband identification No.	LBC *CDH1* variant	Tumor				Structural		Epigenetic, methylation	E-cadherin IHC expression	Other P/LP variants
		Germline *CDH1* variant detected	VAF, %	Somatic *CDH1* variant detected	VAF, %	Variation	iLOH			
834-001	c.1906G>A	Yes	NA	c.163+1G>C	72	Yes	Yes	No	Negative	*PIK3CA*, *ACVR1B*, *CBFB*
834-016	c.2295+16C>A	No	NA	c.511_518delTTTCCTAA	26	Yes	ND	Yes	Negative	*ERBB2*
834-042	c.1462C>T	Yes	NA	c.67C>T	75.3	Yes	Yes	No	Negative	*EPHA3*
834-155	c.1610C>T	Yes	NA	c.532-1G>A	51.2	Yes	ND	No	Negative	*ERBB2*, *ERBB3*
834-163	c.1711+9G>C	NA	NA	NA	NA	NA	NA	NA	Negative	NA
834-183	c.1633C>G	NA	NA	NA	NA	NA	NA	NA	NA	NA
834-190	c.344C>T	Yes	NA	c.2095C>T	46.6	Yes	No	No	Negative	*CBFB*, *GATA3*, *NSD3*, *TP53*, *ZNF703*
834-269	(?_68768071)_(68869950_?)dup	No	NA	c.367C>T	2.4	Yes	No	Yes	Positive	*CCND1*, *FGF19*, *FGF3*, *FGF4*, *TP53*
834-323	c.1633C>G	Yes	NA	c.67C>T	51.8	Yes	Yes	No	Negative	*AKT1*

Abbreviations: IHC, immunohistochemistry; iLOH, intragenic loss of heterozygosity; LB, likely benign; LBC, lobular breast cancer; NA, unavailable tumor sample; ND, not defined (intragenic allelic analysis was not resolutive for iLOH definition); P/LP, pathogenic or likely pathogenic; VAF, variant allele frequency; VUS, variant of unknown significance.

**Figure 2.**
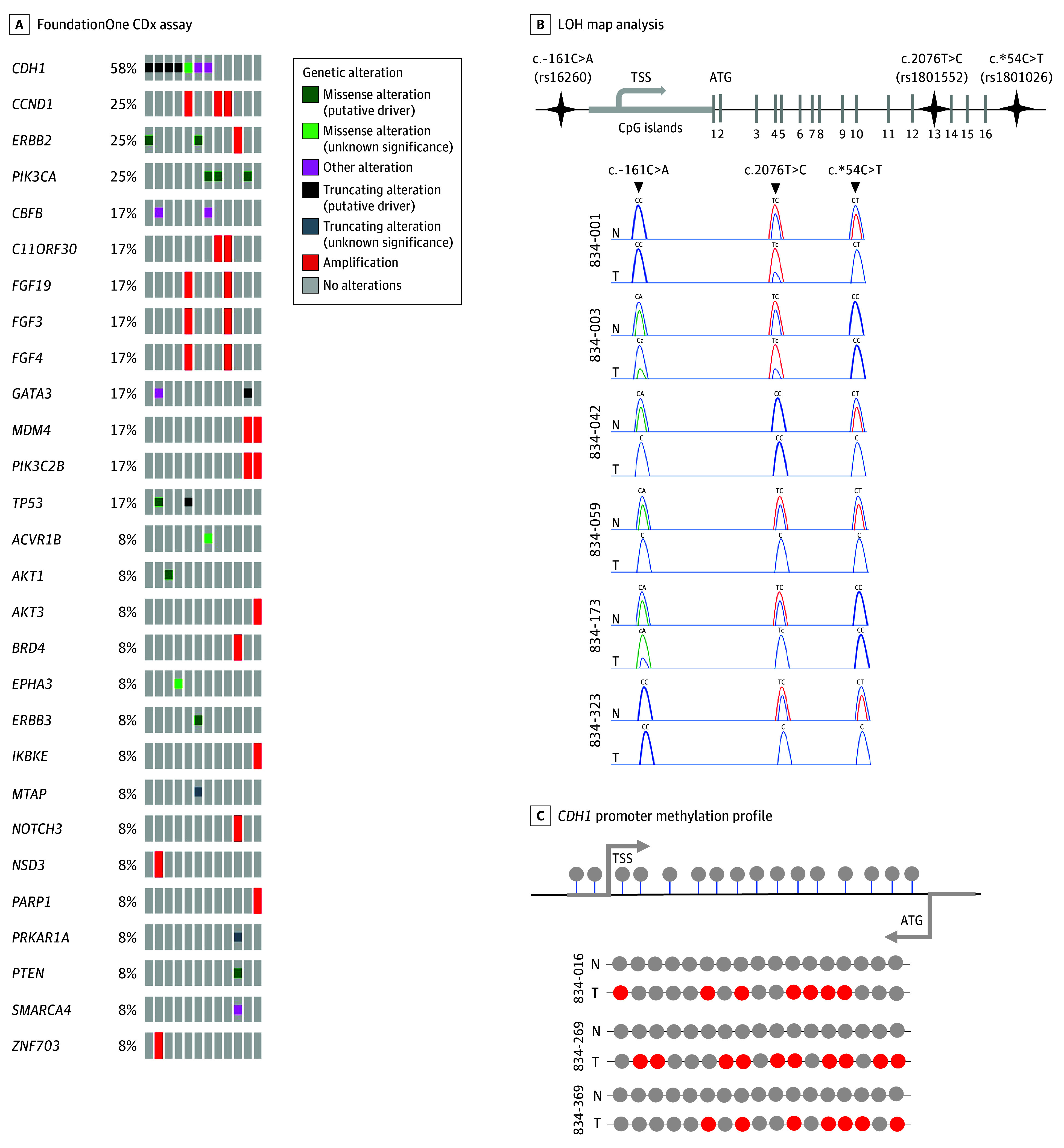
Recurrent Somatic Genomic Alterations in Lobular Breast Cancer Samples From Germline *CDH1* Variant Carriers LOH indicates loss of heterozygosity.

In sporadic LBC (germline *CDH1* VUS or LB), discordances between germline and tumor testing were observed in 2 of 7 cases (28.6%), including 1 germline intronic *CDH1* variant (c.2295+16C>A) and 1 germline *CDH1* copy number variant ([?_68768071]_[68869950_?]dup) missed by tumor sequencing. However, in these 2 cases, somatic *CDH1* variants were detected in tumor samples. A second somatic *CDH1* variant was detected in all LBC samples. Intragenic LOH was detected in 3 of 7 LBCs (42.9%), and promoter methylation was detected in 2 (28.6%) (Table 3) (Figure 2). We noted a high accumulation of genomic aberrations in HLBC samples (Table 2) compared with sporadic LBCs (Table 3): 23 in 5 HLBC groups and 18 in 7 sporadic LBC groups. E-cadherin immunohistochemistry revealed an absent pattern in 12 of 13 analyzed available sample tumors (92.3%) (eFigure 3 in Supplement 1). We observed that epigenetic and genetic *CDH1* somatic alterations occurred only alone in HLBC (Table 2) but simultaneously in sporadic LBC neoplastic lesions (Table 3).

### Clinical Follow-Up

None of the 15 families with LBC who were *CDH1* variant carriers showed a positive first-degree familial history of DGC. Due to the lack of strong evidence in terms of the benefits of prophylactic total gastrectomy (PTG) in the absence of a clear potential gastric cancer (GC) risk, these patients were followed up in accordance with the latest IGCLC recommendations.^7^ For breast surveillance, we suggested annual breast magnetic resonance imaging and mammography, 6-month breast ultrasonography, and annual physical examination for all 15 *CDH1* variant carriers.^1^ With 1 exception, no patients developed DGC during a median of 5.24 years (IQR, 4.93-5.67 years) of follow-up. Conversely, a *CDH1* c.833-476_1138-464del carrier, who was previously tested following a diagnosis of bilateral LBC and then enrolled in this study, had already undergone PTG at enrollment, and gastric histopathological analysis revealed 3 foci of invasive DGC (pT1aN0). Subsequently, this patient also developed a Krukenberg ovary tumor from primary LBC.

### Clinical-Pathological Data

A total of 377 invasive and 17 in situ LBCs were tested for genetic screening. No P/LP variants were detected in the in situ LBCs. With a median 5.24 years of follow-up (IQR, 4.93-5.67 years), no associations were observed among germline *CDH1* variant carriers, *BRCA1* and *BRCA2* genes, wild-type groups, and the clinical-pathological features of LBC (eTables 5 and 6 in Supplement 1). Furthermore, variant carrier or wild-type status was not associated with disease-free survival (eFigure 4 in Supplement 1). Using an age-adjusted Cox proportional hazards regression model, no associations were detected among the explored groups. Exploring the 15 *CDH1* pedigrees, the median age of P/LP *CDH1* variant carriers at LBC diagnosis was significantly lower (42.5 years [IQR, 38.3-43.0 years]) compared with the group carrying VUS plus LB *CDH1* variants (51.0 years [IQR, 45.0-53.0 years]; *P* = .03) and also with the *CDH1* wild-type group (47.0 years [IQR, 43.0-53.0 years]; *P* = .009) (eFigure 1 and eTable 5 in Supplement 1). A lower age at diagnosis was identified in *CDH1* carriers compared with *BRCA1* and *BRCA2* carriers and the wild-type group, but the differences were not significant (eTable 6 in Supplement 1).

## Discussion

### *CDH1* Genetic Testing in HLBC

To our knowledge, this is the largest single-center longitudinal study to report results from germline *CDH1* genetic testing in families with suspected HLBC. In this series, the HLBC phenotype occurred in 34.4% of patients with LBC, whereas a US study reported a lower incidence (15%).^22^ There are some substantial differences between these studies (eTable 8 in Supplement 1). Our study was a European, longitudinal, prospective cohort analysis, and we tested for women with *CDH1* LBC prospectively from phenotype (HLBC) to genotype (*CDH1*). The US study reported a retrospective frequency of approximately 60% of germline *CDH1* variants in its HLBC series. Our frequency of germline *CDH1* variants was substantially lower (3.8%) and seems to be more in accordance with the rarity of this syndrome.

The identification of P/LP germline *CDH1* variants in women with early-onset LBC suggests new criteria for genetic screening selection and recommendations to test this group of patients with LBC. Positive BC family history and juvenile age at LBC diagnosis should be considered to be pivotal criteria for *CDH1* genetic testing in women with LBC. We identified that 83.3% of germline *CDH1* variant carriers had a diagnosis at 45 years or younger and a positive BC family history.

### *BRCA* vs *CDH1*

We demonstrated the mutual exclusion of *BRCA* and *CDH1* genes in the pathway of families with HLBC. A German study suspected this evidence in just 1 pedigree analysis.^23^ In the present HLBC series, we identified that P/LP *BRCA1*, *BRCA2*, and *CDH1* variants occurred with a frequency of 0.2%, 1.2%, and 1.5%, respectively. In cases of sporadic LBC, Yadav et al^8^ reported a frequency of P/LP *BRCA1*, *BRCA2*, and *CDH1* variants of 0.3%, 2.2%, and 0.5%. In an ongoing independent study by our team testing only sporadic LBC (LobularCard Breast trial), the frequency of P/LP variants was 0.9% in *BRCA1* and 2.2% in *BRCA2* genes, and no P/LP *CDH1* variants were identified (eTable 9 in Supplement 1). It seems that there is an association between *CDH1* and HLBC (more than *BRCA1* and *BRCA2*), and this mutual exclusion suggests that *CDH1* HLBC is an inherited cancer predisposition syndrome unrelated to *BRCA*.

### Somatic *CDH1* Alterations, Second Hit, and E-Cadherin Inactivation

In our study of LBC cases associated with germline *CDH1* variants, 11 of 12 (91.7%) manifested a somatic alteration (7 of 12 [58.3%], sequence variations; 6 of 12 [50.0%], intragenic LOH; 3 of 12 [25.0%], promoter methylation)(Table 2 and Table 3). All *CDH1* variant carriers presented with an invasive LBC histotype, thus demonstrating a pivotal role of E-cadherin protein inactivation in the process of lobular breast tumorigenesis. Loss of E-cadherin expression is an early gatekeeper event in in situ LBC and a precursor of invasive LBC.^24,25^

Inactivated sequence variations in the *CDH1* gene have been frequently described as the cause of E-cadherin protein deregulation in invasive LBC.^26,27,28,29^ In this study, all sporadic LBCs had a second structural *CDH1* somatic sequence variation (Table 3); intragenic LOH and methylation were detected in 42.9% and 28.6% of LBCs, respectively. In HLBC series, we were able to identify a second-hit mechanism in 66.7% of analyzed samples matched with identified germline P/LP *CDH1* variants. Different from the HDGC tumors in which *CDH1* promoter methylation appears to be the predominant inactivating mechanism (around 32%),^30^ we rarely observed methylation phenomena in HLBC tumors (Table 2 and eTable 7 in Supplement 1). The difference in genetic and epigenetic mechanisms described between HDGC and HLBC tumors could be a possible explanation for different risk for lobular and gastric tumors in these 2 syndromes.

By exploring FoundationOne data (Table 2 and Table 3), we noted the absence of additional somatic *CDH1* variations, and respective germline variations were confirmed in all HLBC tumors. We noted also that in HLBC tumors, there was a higher accumulation of aberrant variations (Table 2) compared with sporadic LBC (Table 3). We could suppose that this phenomenon is associated with a long progression of *CDH1* HLBC tumorigenesis resulting in more aggressive and undifferentiated tumors.

### BC Risk in Families With HLBC

A recent European study^20^ demonstrated an association of P/LP germline *CDH1* truncating alterations with LBCs, not fulfilling the 2020 HDGC criteria.^7^ However, the exact LBC risk in this nonclassic context is under evaluation; it seems variable in accordance with some different clinical phenotypes.^31,32,33^ Our study demonstrated a significantly earlier age at diagnosis of LBC manifestation in P/LP *CDH1* variant carriers compared with other groups (wild-type, VUS plus B/LB) and also *BRCA1* and *BRCA2* genes. This evidence supports the hypothesis that germline P/LP *CDH1* sequence variations may determine the earliest LBC manifestation in the HLBC phenotype.

### GC Risk in Families With HLBC

The GC risk in asymptomatic P/LP germline *CDH1* variant carriers fluctuates depending on the family history, the age at GC diagnosis, and the number of GC cases in the same family.^31,32,33^ In the context of *CDH1* HLBC, the exact GC risk is unknown. Considering the absence or unclear diagnosis of a positive GC family history, we could consider that the GC risk is presumably lower, but it should not be ignored.^22^ The 2020 IGCLC guidelines reduce the emphasis on PTG if GC family history is weak,^7^ as in HLBC syndrome. In patients not undergoing PTG, endoscopic surveillance remains the unique option.^34^ In our analysis of 15 pedigrees, only 1 documented DGC was reported and none of the remaining 14 women with LBC developed GC within a median 5 years of follow-up. In accordance with the latest IGCLC indications,^7^ the benefits and risks of different preventive options (including PTG) were discussed in a multidisciplinary evaluation.

### Limitations

The following limitations must be considered. First, due to the low frequency of identified germline *CDH1* variants, we were unable to estimate the exact risk for LBC predisposition in families with HLBC. We could assume that the association between the early diagnosis of LBC (age ≤45 years) and P/LP *CDH1* variants correlates with relevant risk for developing LBC. Second, the identified frequency of *CDH1* variants in this study may have been underestimated because we were able to test only 394 women with LBC due to the lack of availability of contacts, patient refusal, and unavailability of informed consent or stored biologic samples. Third, 46.7% of identified germline variants were classified as VUS, and we were not able to assess definitively their pathogenicity. Further studies should explore new predictive tools to solve this relevant issue since the current models are not sufficient to solve this point.

## Conclusions

The data from this cohort study provide a detailed analysis of genotype-phenotype associations in women with HLBC phenotype who have been tested for germline *CDH1* variants. We defined a group of patients (women with LBC with an early age at diagnosis and/or a positive family history for BC) with P/LP germline *CDH1* variants not fulfilling the classic HDGC criteria and with an uncertain risk of developing GC. These data may assist in the genetic counseling on individual risk for families with HLBC carrying P/LP *CDH1* variants, particularly in the absence of germline *BRCA1* and *BRCA2* variants. In addition, our data provide new evidence of *CDH1* second-hit alterations as a main mechanism of HLBC tumorigenesis, suggesting future investigations for the definition of new theranostic biomarkers.

## Notes

Supplement 1. **eTable 1.** Clinical Criteria Adopted in the Study for Germline *CDH1* Genetic Testing in LBC Women **eTable 2.** In Silico Predictions for *CDH1* Missense Variants **eTable 3.** Germline *BRCA1* Variants Identified in Patients With LBC Enrolled in the Study **eTable 4.** Germline *BRCA2* Variants Identified in Patients With LBC Enrolled in the Study **eTable 5.** Median Age at Diagnosis Between Pathogenic vs Other Identified *CDH1* Variants or Wild-Type **eTable 6.** Comparison of Clinical-Pathological Variables in Relation to the Genetic Profile of Patients With LBC **eTable 7.** Overall Frequency of *CDH1* Structural and Epigenetic Alterations Described in Literature and in This Study’s HLBC Tumors **eTable 8.** Differences Between the 2 Studies That Explored *CDH1* Gene Testing in HLBC **eTable 9.** Identified Germline P/LP *BRCA1*, *BRCA2*, and *CDH1* Variants **eFigure 1.** Flow Diagram of the Study Organization, Germline Genetic Testing Results, and Clinical Features of Families With Germline *CDH1* Variant **eFigure 2.** Pedigree With Germline Pathogenic *CDH1* Variation Carriers **eFigure 3.** Loss of E-Cadherin Expression in Invasive Lobular Breast Carcinoma From *CDH1* Germline Variant Carrier **eFigure 4.** Disease-Free Survival Results Between Different Variant Statuses, Including Wild-Type Population

Supplement 2.
Data Sharing Statement
