# Supplementary material for: CDH1 Genotype Exploration in Women With Hereditary Lobular Breast Cancer Phenotype
Source: JAMA Netw Open. 2024 Apr 23;7(4):e247862. doi: 10.1001/jamanetworkopen.2024.7862 (PMC11040411; doi:10.1001/jamanetworkopen.2024.7862)
Supplement: Supplement 1. — eTable 1. Clinical Criteria Adopted in the Study for Germline CDH1 Genetic Testing in LBC Women eTable 2. In Silico Predictions for CDH1 Missense Variants eTable 3. Germline BRCA1 Variants Identified in Patients With LBC Enrolled in the Study eTable 4. Germline BRCA2 Variants Identified in Patients With LBC Enrolled in the Study eTable 5. Median Age at Diagnosis Between Pathogenic vs Other Identified CDH1 Variants or Wild-Type eTable 6. Comparison of Clinical-Pathological Variables in Relation to the Genetic Profile of Patients With LBC eTable 7. Overall Frequency of CDH1 Structural and Epigenetic Alterations Described in Literature and in This Study’s HLBC Tumors eTable 8. Differences Between the 2 Studies That Explored CDH1 Gene Testing in HLBC eTable 9. Identified Germline P/LP BRCA1, BRCA2, and CDH1 Variants eFigure 1. Flow Diagram of the Study Organization, Germline Genetic Testing Results, and Clinical Features of Families With Germline CDH1 Variant eFigure 2. Pedigree With Germline Pathogenic CDH1 Variation Carriers eFigure 3. Loss of E-Cadherin Expression in Invasive Lobular Breast Carcinoma From CDH1 Germline Variant Carrier eFigure 4. Disease-Free Survival Results Between Different Variant Statuses, Including Wild-Type Population [file jamanetwopen-e247862-s001.pdf]

Supplementary Online Content

Corso G, Marino E, Zanzottera C, et al. *CDH1* genotype exploration in women with hereditary lobular breast cancer phenotype. *JAMA Netw Open*. 2024;7(4):e247862. doi:10.1001/jamanetworkopen.2024.7862

eTable 1. Clinical Criteria Adopted in the Study for Germline *CDH1* Genetic Testing in LBC Women

eTable 2. In Silico Predictions for *CDH1* Missense Variants

eTable 3. Germline *BRCA1* Variants Identified in Patients With LBC Enrolled in the Study

eTable 4. Germline *BRCA2* Variants Identified in Patients With LBC Enrolled in the Study

eTable 5. Median Age at Diagnosis Between Pathogenic vs Other Identified *CDH1* Variants or Wild-Type

eTable 6. Comparison of Clinical-Pathological Variables in Relation to the Genetic Profile of Patients With LBC

eTable 7. Overall Frequency of *CDH1* Structural and Epigenetic Alterations Described in Literature and in This Study's HLBC Tumors

eTable 8. Differences Between the 2 Studies That Explored *CDH1* Gene Testing in HLBC

eTable 9. Identified Germline P/LP *BRCA1*, *BRCA2*, and *CDH1* Variants

eFigure 1. Flow Diagram of the Study Organization, Germline Genetic Testing Results, and Clinical Features of Families With Germline *CDH1* Variant

eFigure 2. Pedigree With Germline Pathogenic *CDH1* Variation Carriers

eFigure 3. Loss of E-Cadherin Expression in Invasive Lobular Breast Carcinoma From *CDH1* Germline Variant Carrier

eFigure 4. Disease-Free Survival Results Between Different Variant Statuses, Including Wild-Type Population

This supplementary material has been provided by the authors to give readers additional information about their work.

© 2024 Corso G et al. *JAMA Network Open*.

eTable 1. Clinical criteria for germline *CDH1* genetic testing in LBC women adopted in the study.

Criteria	LBC manifestation	Age at onset	BC family history
A	Bilateral	Any age	Positive/Negative
B	Unilateral	Any age	Positive
C*	Unilateral	≤45	Negative

*Sporadic early onset LBC is a novel criterion; LBC, lobular breast cancer

© 2024 Corso G et al. *JAMA Network Open*.

eTable 2. *In silico* predictions for *CDH1* missense variants. Missense variants identified in this study were evaluated through *in silico* approaches analysing sequence homology, amino acid physical properties, and their impact on protein structure. In detail, the impact of each variant on protein function was estimated through PROVEAN (Protein Variation Effect Analyzer, http://provean.jcvi.org/index.php), SIFT (Sorting Intolerant From Tolerant, http://sift.jcvi.org/), PolyPhen-2 (http://genetics.bwh.harvard.edu/pph2/), and FoldX (http://foldxsuite.crg.eu/) algorithms. PROVEAN, SIFT and Polyphen-2 software were run with the Ensembl transcript 261769, using the following substitutions: T115M, P488S, P537L, R545G, and A636T. PROVEAN classifies variants as deleterious with a score equal or below -2.5. Variants generating a score below 0.05 by SIFT are classified as damaging. Structural impact of specified variants was calculated using FoldX (version 5) implemented in Linux environment (Ubuntu 20.04.2 LTS). Native-state stability changes between mutant and wild-type structures ($\Delta\Delta G = \Delta GMut - \Delta GWT$) were automatically calculated in 5 runs. Mutations with $\Delta\Delta G > 0.8$kcal/mol are considered destabilizing. For our missense variants, scores obtained in each algorithm are specified in the table below. Despite rarely observed, the applied algorithms were consistent for c.1610C>T and c.1906G>A variants. Specifically, the c.1610C>T variant is located in a conserved region and is expected to affect E-cadherin function (PROVEAN score -5.80, SIFT score 0.004, and PolyPhen score 0.999). This variant is also predicted to lead to structural destabilization with an energetic difference between mutant and the WT reference of 1.021 kcal/mol, which may indicate its premature degradation by mechanisms of protein quality control. For the c.1906G>A variant, all the *in silico* tools predict an unlikely functional impact. As observed in table below, PROVEAN, SIFT and PolyPhen yield scores compatible with neutral or tolerated variants. Further, no major impact in protein structure is foreseen for this variant according to FoldX. Concerning the remaining missense variants, potential impact was variable across the distinct tools. SIFT did not identify c.344C>T, c.1462C>T and c.1633C>G as potentially damaging, whereas PolyPhen attributed the highest scores compatible with pathogenicity (0.992, 1.000 and 0.996, respectively). On the other hand, PROVEAN considered c.344C>T as neutral, and c.1462C>T and c.1633C>G as deleterious (scores of -6.07 and -3.22, respectively). Of note, no destabilizing effects were predicted for either variant through FoldX.

**Nucleotide change**	**Protein change**	**Domain**	**PROVEAN**	**SIFT**	**PolyPhen**	**FoldX (kcal/mol)**
c.344C>T	p.(Thr115Met)	Prodomain	Neutral (-1.93)	Tolerated (0.080)	Probably damaging (0.992)	Not destabilized (0,551)
c.1462C>T	p.(Pro488Ser)	EC4	Deleterious (-6.07)	Tolerated (0.173)	Probably damaging (1.000)	Not destabilized (0,656)
c.1610C>T	p.(Pro537Leu)	EC4	Deleterious (-5.80)	Damaging (0.004)	Probably damaging (0.999)	Destabilized (1,021)
c.1633C>G	p.(Arg545Gly)	EC4	Deleterious (-3.22)	Tolerated (0.159)	Probably damaging (0.996)	Not destabilized (0,706)
c.1906G>A	p.(Ala636Thr)	EC5	Neutral (-0.45)	Tolerated (0.571)	Benign (0.173)	Not destabilized (0,200)

© 2024 Corso G et al. *JAMA Network Open*.

eTable 3. Germline *BRCA1* variants identified in LBC patients enrolled in the study.

ID	Sequence variant *	Protein change	Type	Interpretation
834-034	c.3835G>A	p.(Ala1279Thr)	Missense	Likely Benign
834-047	c.3116C>T	p.(Ala1039Val)	Missense	VUS
834-074	c.3257T>G	p.(Leu1086Ter)	Nonsense	Pathogenic
834-088	c.3596C>T	p.(Ala1199Val)	Missense	Likely Benign
834-162	c.509G>A	p.(Arg170Gln)	Missense	VUS
834-167	c.2933A>G	p.(Tyr978Cys)	Missense	VUS
834-178	c.43A>C	p.(Ile15Leu)	Missense	Likely Benign
834-316	c.1251T>G	p.(Asn417Lys)	Missense	VUS
834-373	c.535T>C	p.(Tyr179His)	Missense	VUS

* HGVS nomenclature [Reference sequence (Human Feb. 2009 - GRCh37/hg19 Assembly): NM_007294.4];
VUS, variant of unknown significance; LBC, lobular breast cancer

© 2024 Corso G et al. *JAMA Network Open*.

eTable 4. Germline *BRCA2* variants identified in LBC patients enrolled in the study.

ID	Sequence variant *	Protein change	Type	Interpretation
834-018	c.7507G>A	p.(Val2503Ile)	Missense	VUS
834-031	c.1256G>A	p.(Cys419Tyr)	Missense	VUS
834-031	c.10154G>A	p.(Arg3385His)	Missense	VUS
834-045	c.9271G>A	p.(Val3091Ile)	Missense	VUS
834-061	c.5267T>A	p.(Val1756Glu)	Missense	VUS
834-081	c.7180A>T	p.(Arg2394Ter)	Nonsense	Pathogenic
834-083	c.2808_2811delACAA	p.(Ala938ProfsTer21)	Frameshift	Pathogenic
834-144	c.7007+16A>C	(p.?)	Intronic	VUS
834-150	c.518G>T	p.(Gly173Val)	Missense	VUS
834-184	c.235A>G	p.(Ile79Val)	Missense	VUS
834-193	c.3310A>C	p.(Thr1104Pro)	Missense	VUS
834-193	c.3503T>A	p.(Met1168Lys)	Missense	VUS
834-197	c.5217_5220del	p.(Tyr1739Ter)	Nonsense	Pathogenic
834-223	c.4339G>A	p.(Val1447Ile)	Missense	VUS
834-246	c.64G>A	p.(Ala22Thr)	Missense	VUS
834-261	c.9101A>G	p.(Gln3034Arg)	Missense	VUS
834-290	c.6523G>C	p.(Glu2175Gln)	Missense	Likely Benign
834-336	c.3413A>T	p.(Gln1138Leu)	Missense	VUS
834-340	c.7558C>T	p.(Arg2520Ter)	Nonsense	Pathogenic
834-343	c.2905C>T	p.(Gln969Ter)	Nonsense	Pathogenic
834-348	c.6405_6409del	p.(Asn2135LysfsTer3)	Frameshift	Pathogenic
834-361	c.6461A>C	p.(Tyr2154Ser)	Missense	VUS
834-392	c.4301A>T	p.(Lys1434Ile)	Missense	VUS
834-399	c.6037A>G	p.(Lys2013Glu)	Missense	VUS

* HGVS nomenclature [Reference sequence (Human Feb. 2009 - GRCh37/hg19 Assembly): NM_000059.4]
VUS, variant of unknown significance; LBC, lobular breast cancer

© 2024 Corso G et al. *JAMA Network Open*.

eTable 5. Median age at diagnosis between pathogenic vs other identified *CDH1* variants/wild-type.

**a**	**P**	**VUS+LB**	***P*-value for difference**
			**.03**
**Age, median (IQR)**	42.5 (38.3-43.0)	51 (45.0-53.0)	

**b**	**P**	**VUS+LB+WT**	***P*-value for difference**
			**.009**
**Age, median (IQR)**	42.5 (38.3-43.0)	47 (43.0-53.0)	

© 2024 Corso G et al. *JAMA Network Open*.

eTable 6. Comparison of clinical-pathological variables in relation to the genetic profile of patients with LBC.

Variable	Overall (394)	Wild-type (350)	*CDH1* (15)	*BRCA1* (9)	*BRCA2* (22)	*P*-value
Age, median (IQR)	46 (43-53)	47 (43-53)	44 (42.5-51)	48 (44.5-54.5)	46.5 (41-55.8)	0.61
Menopausal status						
Pre-	239 (60.7)	214 (61.1)	8 (53.3)	6 (66.7)	12 (54.5)	
Post-	117 (29.7)	100 (28.6)	6 (40.0)	3 (33.3)	9 (40.9)	.36
Peri-	34 (8.6)	34 (9.7)	0 (0)	0 (0)	0 (0)	
Missing	4 (1.0)	2 (0.6)	1 (6.7)	0 (0)	1 (4.6)	
BC family history						
No	124 (31.5)	108 (30.9)	5 (33.3)	2 (22.2)	9 (40.9)	
1st degree	138 (35.0)	127 (36.3)	3 (13.3)	5 (55.6)	5 (22.7)	
2nd degree	126 (32.0)	110 (31.4)	6 (40.0)	2 (22.2)	8 (36.4)	.83
3rd degree	1 (0.2)	1 (0.3)	0 (0)	0 (0)	0 (0)	
1st and 2nd degree	1 (0.2)	1 (0.3)	0 (0)	0 (0)	0 (0)	
Missing	4 (1.1)	3 (0.8)	1 (6.7)	0 (0)	0 (0)	
Grade						
1	45 (11.4)	39 (11.1)	1 (6.7)	1 (11.1)	4 (18.2)	
2	266 (67.5)	234 (66.9)	12 (80.0)	6 (66.7)	14 (63.6)	.46
3	57 (14.5)	54 (15.4)	0 (0)	2 (22.2)	3 (13.6)	
Missing	26 (6.6)	23 (6.6)	2 (13.3)	0 (0)	1 (4.6)	
pT						
pT1	191 (48.5)	168 (48.0)	8 (53.5)	5 (55.6)	11 (50)	
pT2-pT4	178 (45.2)	157 (44.9)	7 (46.5)	4 (44.4)	11 (50)	.91
pTis	16 (4.1)	16 (4.6)	0 (0)	0 (0)	0 (0)	
Missing	9 (2.2)	9 (2.5)	0 (0)	0 (0)	0 (0)	
pN						
pN0	97 (24.6)	87 (24.9)	3 (20)	3 (33.3)	5 (22.7)	
pN+	292 (74.1)	259 (74.0)	11 (73.3)	6 (66.7)	17 (77.3)	.99
Missing	5 (1.3)	4 (1.1)	1 (6.7)	0 (0)	0 (0)	
HER2						
Negative	338 (85.8)	302 (86.3)	12 (80)	8 (88.9)	18 (81.8)	
Positive*	16 (4.1)	14 (4.0)	1 (6.7)	0 (0)	1 (4.5)	.99
Unknown/doubt	40 (10.1)	34 (9.7)	2 (13.3)	1 (11.1)	3 (13.7)	
Ki67, median (IQR)	15 (10-20)	15 (10-20)	16 (13.5-21.5)	10 (10-10.5)	17.5 (10.22.8)	.13
ER, median (IQR)	95 (90-95)	95 (90-95)	95 (90-95)	92.5 (90-95)	95 (90-95)	.90
PgR, median (IQR)	90 (60-95)	90 (60-95)	80 (72.5-95)	90 (27.5-92.5)	90 (62.5-95)	.97

*HER2 3+, or HER2 2+ with amplified FISH

LBC, lobular breast cancer; IQR, interquantile range; HER2, human epidermal growth factor receptor-2, ER, estrogen receptor, PgR, progesterone receptor.

© 2024 Corso G et al. *JAMA Network Open*.

eTable 7. Overall frequency of *CDH1* structural and epigenetic alterations described in literature and in our HLBC tumors.

	Structural		Epigenetic
	**Mutation**	**LOH**	**Methylation**
Sporadic DGC	4.5%	4.5%	25%
Sporadic LBC	15-56%	50%	41-53%
HDGC	3%	14%	47%
HLBC	0	60%	20%

LOH, loss of heterozygosity; DGC, diffuse gastric cancer; LBC, lobular breast cancer; HLBC, hereditary lobular breast cancer, HDGC, hereditary diffuse gastric cancer.

© 2024 Corso G et al. *JAMA Network Open*.

eTable 8. Differences between the two studies that explored *CDH1* gene testing in HLBC.

	HLBC families	Positive *CDH1* variants	Negative *CDH1* variants	PTG	GC after PTG	LBC clinic-pathological, survival data	Second-hit characterization	Method
**American study**	31	19 (61.3%)	Unknown	16	15	No	No	Prospective cross sectional cohort
**European Study**	394	15 (3.8%)	379	1	1	Yes	Yes	Prospective longitudinal cohort

© 2024 Corso G et al. *JAMA Network Open*.

eTable 9. Identified germline P/LP *BRCA1/2* and *CDH1* variants.

Gene	Sporadic Lobular BC*	Sporadic Ductal BC*	Sporadic LBC (our series)**	HLBC (our series)***	HBOC****
BRCA1	0.3%	2,3%	0.9%	0.2%	10-20%
BRCA2	2,2%	2,4%	2.2%	1.2%	5-10%
CDH1	0.5%	0.04%	0%	**1.5%**	Not reported

*Yadav et al. JCO 2021
**Unpublished data extracted from an independent study still running in our cancer institute (LobularCard Breast trial)
***Current study
****Other data extracted from literature

© 2024 Corso G et al. *JAMA Network Open*.

eFigure 1. CONSORT flow-diagram of the study organization, germline genetic testing results, and clinical features of families with germline *CDH1* variant. After initial selection of 5429 primary LBC women, we selected 1867 patients with "expanded" HLBC clinical criteria. A total of 421 LBC women were enrolled, of which 394 were actually tested. A total of 48 germline *CDH1, BRCA1*, *or BRCA2* variants were detected. Pedigrees of *CDH1* variant-carriers were explored, with 437 relatives and 116 cancer phenotypes, and BC was the most frequently identified tumor.

* Probands or relatives can contribute more than one phenotype.

& Mean age at diagnosis.

FH: family history; LBC: lobular breast cancer; HLBC: hereditary lobular breast cancer; P/LP: pathogenic/likely pathogenic; VUS: variant of unknown significance; LB: likely benign; BC: breast cancer.

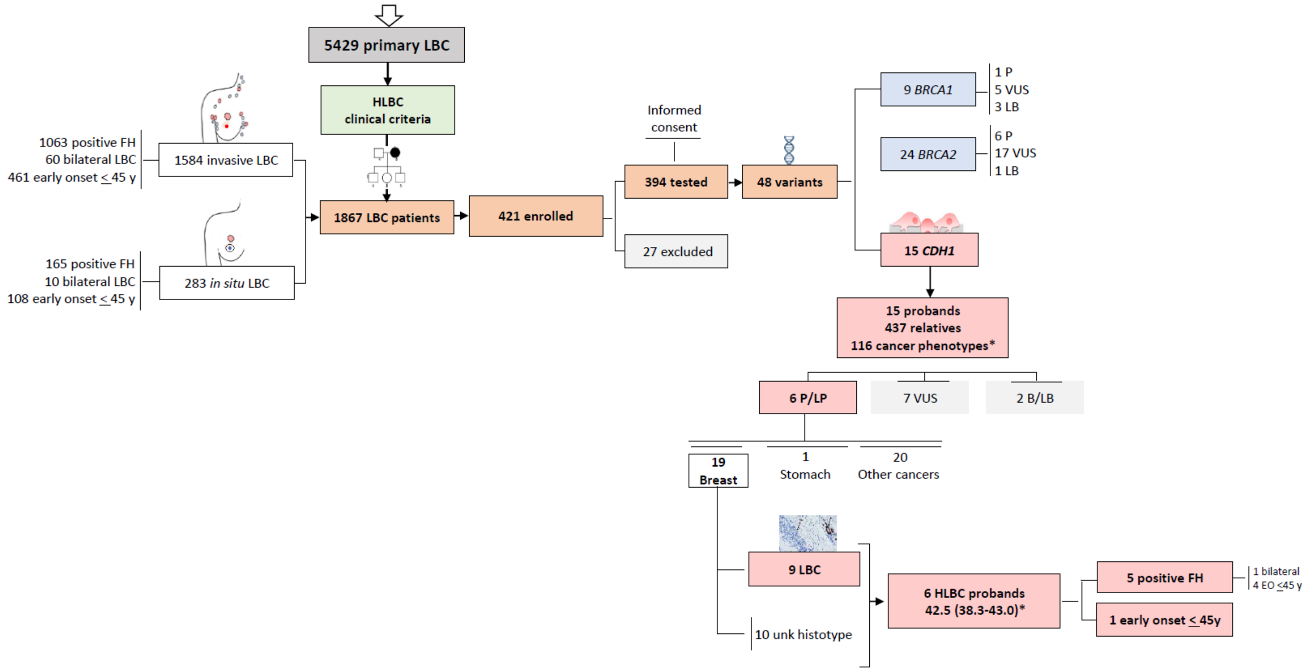

© 2024 Corso G et al. *JAMA Network Open*.

eFigure 2. Pedigree with germline *CDH1* mutations (pathogenic) carriers. Arrow indicate the probands, blue symbols (up-right) breast cancer.

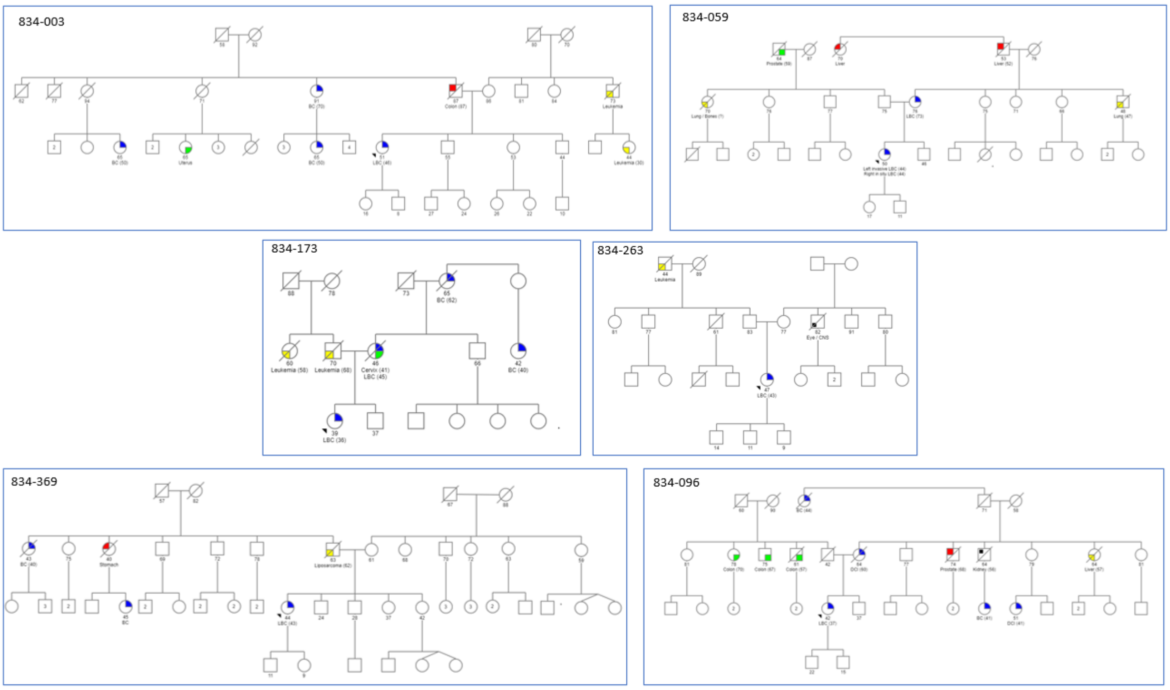

© 2024 Corso G et al. *JAMA Network Open*.

eFigure 3. Loss of E-cadherin expression in invasive lobular breast carcinoma from *CDH1* germline variant carrier. Non-to-poorly cohesive neoplastic elements of classic invasive LBC (A, Hematoxylin and eosin, original magnification 100x) showing no immunoreactivity for E-cadherin (B); breast duct (B, arrow) colonized by pagetoid spreading E-cadherin-negative neoplastic cells with a residual inner layer of E-cadherin-positive normal luminal epithelial cells (E-cadherin immunohistochemistry, original magnification 200x).

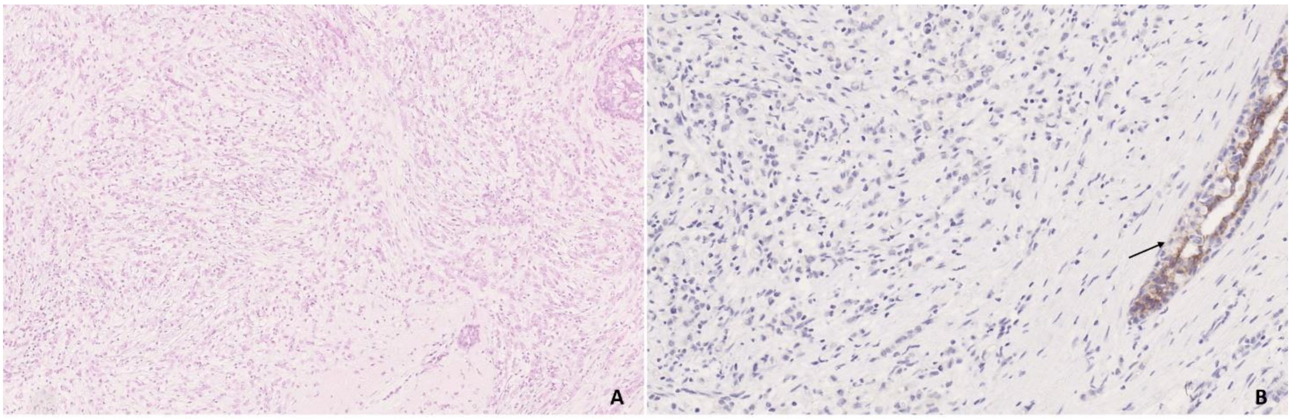

© 2024 Corso G et al. *JAMA Network Open*.

eFigure 4. Disease-free survival results between the different variant status, including wild-type population.

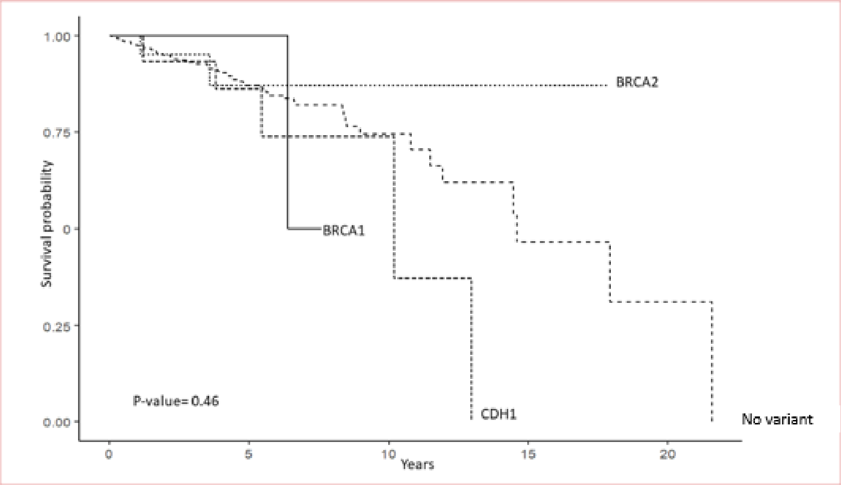

© 2024 Corso G et al. *JAMA Network Open*.
