# Supplementary material for: CDH1 Genotype Exploration in Women With Hereditary Lobular Breast Cancer Phenotype
Source: JAMA Netw Open. 2024 Apr 23;7(4):e247862. doi: 10.1001/jamanetworkopen.2024.7862 (PMC11040411; doi:10.1001/jamanetworkopen.2024.7862)
Supplement: Supplement 2. — Data Sharing Statement [file jamanetwopen-e247862-s002.pdf]

Data Sharing Statement

Corso. CDH1 Genotype Exploration in Women With Hereditary Lobular Breast Cancer Phenotype. *JAMA Netw Open*. Published April 23, 2024.
doi:10.1001/jamanetworkopen.2024.7862

Data
Data available: No

Additional Information
Explanation for why data not available: Data contains sensitive information
